# The effect of community dialogues and sensitization on patient reporting of adverse events in rural Uganda: Uncontrolled before-after study

**DOI:** 10.1371/journal.pone.0203721

**Published:** 2019-05-09

**Authors:** Helen Byomire Ndagije, Leonard Manirakiza, Dan Kajungu, Edward Galiwango, Donna Kusemererwa, Sten Olsson, Anne Spinewine, Niko Speybroeck

**Affiliations:** 1 National Pharmacovigilance Centre, National Drug Authority, Kampala, Uganda; 2 Makerere University Centre for Health and Population Research, Iganga, Uganda; 3 Sten Olsson Pharmacovigilance Consulting, Uppsala, Sweden; 4 Institute of Health and Society (IRSS), Université Catholique de Louvain, Brussels, Belgium; University of Sydney, AUSTRALIA

## Abstract

**Background:**

Patients experiencing adverse drug events (ADE) in many developing countries are in the best position to report these events to the authorities but need to be empowered to do so. Systematic evaluation of community engagement and patient support especially in rural areas would provide evidence for a program to monitor potential harm from medicines. The aim of this study was to assess the effects of a community dialogue and sensitization (CDS) program on the knowledge, attitude and practises of community members for reporting ADE.

**Methods:**

This an uncontrolled before-after study was conducted in two eastern Ugandan districts between September 2016 and August 2017

**Results:**

After implementation of the community dialogue and sensitization (CDS) program, there was an overall 20% (95% CI:16% to 25%) increase in knowledge about ADE in the community compared to before the program began. Awareness levels increased by 50% (95% CI: 37% to 63%) among those with little or no education and by41% (95% CI: 31% to 52%) among young people (15–24 years). Furthermore, 5% (95% CI: 3% to 7%) more respondents recognized the need for reporting ADEs compared to before the program. Finally, there was a significant increase of 115% (95% CI:137% to 217%) in respondent recognition and reporting of ADEs compared to the beginning of the CDS program. Overall, this community found the CDS program acceptable and proposed aspects that could be improved for future use.

**Conclusion:**

Our evaluation showed that the CDS program increased knowledge and improved attitudes by catalyzing discussions among community members and healthcare professionals on health issues and monitoring safety of medicines compared to before the program. Successful implementation of the program depends on holistic health systems strengthening and adaptation to the community’s way of life.

## Introduction

Globally, adverse drug reactions account for up to 18% of hospital deaths [1–5]. An adverse drug event (ADE) is defined as any negative or harmful occurrence that takes place during treatment that may or may not be associated with a medicine [6]. Uganda uses spontaneous reporting of suspected ADEs by predominately healthcare professionals to monitor the safety of medicines [7]. While Ugandan pharmacovigilance regulations from 2014 require healthcare professionals to report ADEs [6], the grossly under-resourced health sector makes effective reporting difficult [8]. A recent study reported an incidence of 25% of hospital-acquired suspected ADEs among Uganda inpatients [9]. Antibiotics and anti-malarials are the most commonly implicated drugs in community-acquired ADEs [10]. With increased access and use of medicines in the community, it is becoming increasingly important to collect more safety information by involving patients directly. Community health extension workers (CHEWs) have minimized the role of healthcare professionals in the delivery of healthcare in the community [11, 12]. Healthcare professionals have insufficient knowledge of existing ADE reporting systems [13]. As ADE reporting is important to improving healthcare delivery, the National Pharmacovigilance Centre (NPC) intends to implement a program that encourages community members to directly report possible ADEs and other drug related events.

Community dialogue and sensitization (CDS), is a program designed to stimulate community support and engagement in improving health-seeking behaviour. This program builds on existing community-based structures using regular dialogues and sensitization sessions through exploring relevant health topics, identifying issues and using this information for planning for improved health service delivery. A community dialogue and sensitization (CDS) program was adopted from a similar approach in other African countries [14]. The program added a sensitization campaign using radio messages, posters and brochures to raise drug-safety awareness, encourage dialogue, and involve the community in designing pertinent solutions. This approach involved a participatory communications process of sharing information through existing community-based structures. It was aimed at enabling communities to make informed choices and to take individual and collective action.

The aim of this paper was to assess the impact of a CDS program on the knowledge, attitudes and practices (KAPs) of community members for reporting ADEs in two eastern Ugandan districts.

## Materials and methods

### Study design

This uncontrolled before-after study was conducted between September 2016 and August 2017 in the Iganga/Mayuge Health Demographic Surveillance Site (IMHDSS) of Uganda. Prior to the CDS program in September 2016 and the end-line survey in July 2017, we conducted a representative, cross-sectional household baseline survey.

### Setting

The CDS program was implemented in two predominantly rural districts of Iganga and Mayuge in eastern Uganda. The NPC and Makerere University Centre for Health and Population Research (MUCHAP) jointly developed the CDS toolkit. It included a facilitator guide-book with ten steps, key talking points, pictorial posters, and a monitoring and feedback tool. Tools and images were pre-tested with the target audience before finalizing the toolkit. The CDS program included several components (see Table 1) conducted between December 2016 and April 2017 (four months).

**Table 1:** Multiple program interventions that were used to improve patient reporting.

Program Components	Description	Beneficiaries		Deliverers
		Individual	Others	
**Community dialogue meetings**	**Mobilization**: The team of CHEW &MUCHAP information officers enlisted the support of district health offices and the local council leaders. The LC provided access to the community. Public announcements about the CDS were made in churches, mosques, village meetings & women groups a day prior to the meeting. **Program activities:** The CHEW &MUCHAP information officers conducted the CDS using the toolkit containing the Facilitator’s 10-step process, ADE pictorial posters and brochures in the local language, Lusoga. Forty CDS sessions were conducted January to April 2017 **Reach:** 658 participants (139 men, 519 women).	Primary household member in charge of health care decisions,	Community members Community leaders	CHEW MUCHAP information officer
**Radio messages**	Radio spot messages aimed at raising community awareness were aired 3 times a day (8am, 1pm & 9pm) for three days in a week (Monday, Wednesday & Friday). Period of airing: January to April 2017 The messages were developed by NDA & MUCHAP	Patients and care-givers in the community	Community members, district, religious & village leaders, private &public health service providers	3 radio stations NBS FM, R FM & Safari FM covering Iganga and Mayuge districts
**Focus Group discussions**	FGDs in public health facilities and FGDs, which included health workers and some community members	Community members	Private & public health care providers,	MUCHAP information officer
**Brochures**	Distributed 2000 Lusoga brochures to participants in their households and communities with information about ADEs and encouraged reporting of suspected ADEs during the HDSS routine data collection by field assistants	Household members	Community members	MUCHAP field assistants
**ADE posters**	Distributed 500 posters to private and public health facilities encouraging ADE reporting with some guidelines of how and where to report them	Health workers	Community members Private & public health facilities	MUCHAP staff
**ADE reporting forms**	Distribution of 70 booklets of 25 ADE carbonated reporting forms was accompanied by sensitization of health workers about pharmacovigilance	Health workers in Iganga &Mayuge	Health workers beyond implementation districts	MUCHAP staff NDA staff
**Advocacy**	An official launch of the CDS approach to create more aware ness about the well-being of the community and the importance of ADE reporting. Posters and brochures were also distributed, 16^th^ December 2016 Courtesy call to the district health office by NDA officials	Not applicable	District health authorities Community leaders	NDA staff MUCHAP staff Jounalists

CHEW = Community Health Extension Worker, MUCHAP = Makerere University Centre for Health and Population Research, ADE = Adverse Drug Event, NDA = National Drug Authority, CDS = Community Dialogue and Sensitization, LC = Local Council.

Interviews were carried out by local field researchers using a pre-tested, structured and validated questionnaire translated in the local language (Lusoga) using established guidelines [15]. Field researchers and supervisors attended a two-day training course covering data collection, field procedures and interview techniques. Field supervisors received an additional day of training focusing on supervision of field teams, as well as sampling procedures. Following the pilot study, a half-day training session was conducted to discuss challenges identified during the pre-test. Training materials were prepared by MUCHAP and the National Drug Authority (NDA) while the training was conducted by MUCHAP, responsible for coordination and supervision during field work.

### Participants

Sampling was single-stage and conducted at the household level. In each of the 65 villages in the IMHDSS surveillance area, the MUCHAP study team sampled an equal number of households using a simple random sampling approach with the help of community leaders. A household was defined as a group of people who routinely lived and ate together. One person per selected household was interviewed and chosen based on ability to answer questions on their household’s health. Community members were interviewed using the community members’ questionnaire (S1 File). All community-based health facilities in the program area were included in the survey. At least two healthcare professionals from each health facility were randomly selected and interviewed using a specific healthcare professionals questionnaire (S2 File). Health facilities were both privately and publicly owned and included hospitals, health centers VI-II, pharmacies, drug shops and shops that sell drugs alongside other merchandise.

### Variables

The primary outcomes of this study included participant KAPs towards ADE reporting before and after the CDS program.

The secondary outcome of this study was to establish the acceptability of the CDS program and the best practices for community engagement. Acceptability was defined as the extent to which the program or its attributes were sufficiently tolerable to its users, as reflected in both program uptake and perceived quality.

### Data sources

We measured the percentage difference between respondents’ KAPs towards ADE reporting before and after the CDS program.

Knowledge was measured based on responses to the question “Do drugs “cause” negative or side effects?” For respondents who answered “yes,” their attitude towards reporting ADEs was measured by asking if they considered it necessary to report such events. To understand respondent practices, we asked if they would report any ADE, if encountered.

### Bias

Random sampling was used so that all community members had equal chance of participating in the study to minimize recruitment bias. Also, research assistants made efforts to interview the household head or eldest person to minimize response bias.

### Study size

The entire adult population of the study area was considered as the sampling domain, where all households were eligible for selection. A total sample size of 778 respondents for each survey was determined to allow for the calculation of risk difference before and after the program. Assuming a baseline proportion of ρ = 0.5 and testing at the 0.05 level, a sample size of 389 respondents for each survey was needed for 80% power to detect a change of at least 10%in the primary outcome [16]. Adjusting for confounders, no-response and design effect, we doubled the sample size.

### Quantitative variables

In order to measure community members’ KAPs towards reporting ADEs, the same questions were asked in baseline and end-line surveys. Furthermore, the percentage difference before and after the program assessed for significance using the difference in proportions method.

### Qualitative variables

We conducted interviews with community members and healthcare professionals in the study area after community dialogue meetings and focus group discussions (FGDs).During these meetings, an exploratory research strategy was used to examine attributes of the CDS program that could foster or hinder its uptake within communities. FGDs sought to explore the general trends in communities with respect to CDS, whereas the interviews explored individual experiences. The information was collected using semi-structured interview guidelines (S3 File). Each meeting lasted between one and two hours and all interviews were recorded, transcribed and translated. Notes were inserted in relevant sections of the transcripts to clarify the context in which statements were made, as well as to clarify statements resulting from poor sound quality.

### Statistical methods

Data entry was done using EpiData 3.1 (EpiData Association) software by ten trained data entry officers. All records were double entered to ensure accuracy. Data were transferred to STATA Version 12 (StataCorp LP) for analysis. The relative percentage difference in the responses before and after the program were analyzed using the difference in proportions method. All comparisons were done at a 5% level of significance with 95% confidence intervals for the mean difference in responses before and after the program.

The qualitative data was transcribed, and transcripts were loaded into NVivo 12 for thematic content analysis. Researchers worked together in the same room to discuss possible interpretations, meanings and the most accurate interpretation of derived quotations. Attention was paid to words, phrases and actions of participants that may capture the meaning of what they did or said.

### Ethics approval and consent to participate

The study protocol was approved by the Mildmay Uganda research and ethics committee (REC REF 0604–2017) and Uganda National council of Science and Technology (HS 2247), the institution responsible for national research clearance in accordance with the World Medical Association Helsinki Declaration. Permission to conduct the study in the area was also obtained from NDA, the local administration of Iganga and Mayuge Districts, IMHDSS and MUCHAP. Written informed consent was obtained from the participants.

## Results

### Description of survey respondents

There were 1,034 household adult members in the baseline survey and 827in the end-line survey. All recruited respondents consented to participate. Overall, there was a large positive difference in the KAPs of respondents towards reporting ADEs before and after the CDS program. Table 2 summarizes survey respondent profiles in terms of age, education, religion and occupation.

**Table 2:** Social and demographic characteristics of study participants.

	Before n (%) N = 1034	After n (%) N = 827	Total population
**Age group**			
15–24	163 (15.8)	137(16.6)	21809 (43.0)
25–34	304 (29.4)	224(27.1)	11691(23.1)
35–44	243 (23.5)	200(24.2)	7560 (14.9)
45–54	145 (14.0)	138(16.7)	4966 (9.8)
55–64	105 (10.2)	54(6.5)	2390 (4.7)
65+	74 (7.2)	74(9.0)	2270 (4.5)
**Education**			
None	147 (14.2)	90(10.9)	3279 (6.5)
Primary	532 (51.5)	422(51.0)	28592 (56.4)
Secondary	310 (29.9)	265(32.0)	15703 (31.0)
Tertiary	23 (2.2)	27(3.3)	2048 (4.0)
University	22 (2.1)	23(2.8)	1064 (2.1)
**Religion**			
Anglican	246 (23.8)	251(30.4)	16431 (32.4)
Roman Catholic	93 (9.0)	82(9.9)	4004 (7.9)
Pentecostal	103 (10.0)	103(12.5)	2448 (4.8)
Muslim	573 (55.4)	380(46.0)	27302 (53.9)
Other	19 (1.8)	10(1.2)	502 (1.0)

### Knowledge about ADEs

After implementation of the CDS program, there was an overall 20% (95% CI: 16% to 25%) increase in knowledge about ADEs in the community. Furthermore, there was a percentage change of 41.1% in the knowledge about ADEs among young people aged 15 to 24 years (95% CI:31%to52%) and 50% among the respondents with little or no education (95% CI:37% - 63%) (Table 3).

**Table 3:** Comparison of responses to the question of drugs causing ADEs before and after CDS program.

Do drugs “cause” negative or side effects?												
	Yes				No				Don’t know			
	Before (%)	After (%)	% diff.	95% CI	Before (%)	After (%)	% diff.	95% CI	Before (%)	After (%)	% diff.	95% CI
**Age group**												
15–24	86 (52.8)	102 (74.5)	41.1	31.0 to 52.0	63 (38.7)	33(24.1)	-37.7	-57 to -19	14(8.6)	2(1.5)	-82.6	-122.0 to -43.0
25–34	173 (56.9)	151 (67.4)	18.5	10.0 to 27.0	110(36.2)	67(29.9)	-17.4	-32 to -3	21(6.9)	6(2.7)	-60.9	-82.0 to -39.0
35–44	145 (59.7)	137 (68.5)	14.7	6.0 to 24.0	84(34.6)	55(27.5)	-20.5	-36 to -5	14(5.8)	8(4.0)	-31.0	-50.0 to -12.0
45–54	79 (54.5)	87 (63.5)	16.5	5.0 to 28.0	51(35.2)	43(31.4)	-10.8	-30 to 8	15(10.3)	7(5.1)	-50.5	-76.0 to -25.0
55–64	54 (51.4)	37 (68.5)	33.3	18.0 to 49.0	37(35.2)	13(24.1)	-31.5	-59 to -4	14(13.3)	4(7.4)	-44.4	-80.0 to -8.0
65+	45 (60.8)	46 (62.2)	2.3	-13.0 to 18.0	26(35.1)	25(33.8)	-3.7	-30 to 22	3(4.1)	3(4.1)	0.0	-32.0 to 32.0
**Education**												
None	50 (34.0)	46 (51.1)	50.3	37.0 to 63.0	79(53.7)	37(41.1)	-23.5	-43 to -4	18 (12.2)	7(7.8)	-36.1	-0.6 to -0.1
Primary	294 (55.3)	273 (64.8)	17.2	11.0 to 23.0	191(35.9)	132(31.4)	-12.5	-23 to -2	47(8.8)	16(3.8)	-56.8	-72.0 to -42.0
Secondary	199 (64.2)	193 (72.8)	13.4	6.0 to 21.0	95(30.6)	65(24.5)	-19.9	-34 to -6	16(5.2)	7(2.6)	-50.0	-68.0 to -32.0
Tertiary	19 (82.6)	25 (92.6)	12.1	-6.0 to 30.0	4(17.4)	2(7.4)	-57.5	-109 to -6	0(0.0)	0(0.0)	0.0	0.0
University	20 (90.9)	23(100)	10.0	-2.0 to 22.0	2(9.1)	0(0.0)	-100.0	-140 to -60	0(0.0)	0(0.0)	0.0	0.0
**Religion**												
Anglican	140 (56.9)	174 (69.3)	21.8	13.0 to 30.0	91(37)	69(27.5)	-25.7	-40 to -11	15(6.1)	8(3.2)	-47.5	-66.0 to -29.0
Catholic	59 (63.4)	56 (68.3)	7.7	-6.0 to 22.0	29(31.2)	23(28.0)	-10.3	-35 to 15	5(5.4)	3(3.7)	-31.5	-62.0 to -1.0
Pentecostal	65 (63.1)	77 (74.8)	18.5	6.0 to 31.0	30(29.1)	25(24.3)	-16.5	-40 to 7	8(7.8)	1(1.0)	-87.2	-140.0 to -34.0
Muslim	308 (53.8)	244 (64.2)	19.3	13.0 to 26.0	217(37.9)	118(31.1)	-17.9	-28 to -7	48(8.4)	18(4.7)	-44.1	-58.0 to -30.0
Other	10 (52.6)	9 (90.0)	71.1	42.0 to 100.0	4(21.1)	1(10.0)	-52.6	-124 to 19	5(26.3)	0(0.0)	-100.0	0.0
**Overall**	**582 (56.3)**	**560 (67.8)**	20.4	16.0 to 25.0	**371(35.9)**	**236(28.6)**	**-0.2**	**-28 to -13**	**81(7.8)**	**30(3.6)**	**-0.5**	-64.0 to -43.0

ADE = Adverse Drug Event, DK = Don’t Know, CI = Confidence Interval

The community demonstrated knowledge of what ADEs were as respondents shared their experiences about drugs. When a drug reaction occurred, they considered it the drug’s normal mode of action, and thus did not take it seriously.

*“My child had convulsions and was treated with quinine injection but became crippled …*, *but I knew that it was how that medicine works*.*” (FGD female*, *34 –Buwaiswa)*

Some respondents were able to differentiate the disease symptoms from the effects of the drug, based on how they felt before taking it and the long-term effects of the drug:

*“I used injection for family planning for two months and I started bleeding severely and felt like going to the radio to seek assistance*. *Family planning makes some people huge and others small in size*. *Personally*, *I was small but now am fat*.” (FGD female, 35 –Bukwaya)

However, some respondents could not differentiate between the drug and the disease:

*“I used Coartem and I felt worse than before*. *Too much headache and vomiting*.*”(FGD male 22*, *Buwaiswa)*

### Attitudes towards reporting ADEs

After implementation of the CDS program, there was an overall increase of 5% (95% CI: 3% to 7%) of respondents who considered ADE reporting necessary. Table 4 presents differences in attitudes following the program based on socio demographic characteristics.

**Table 4:** Comparison of study participant attitudes towards the need to report ADEs before and after CDS program^α^.

Necessity to report ADEs				
Yes				
	Before (%)	After (%)	% relative diff.	95%CI
**Age category**				
15–24	144 (88.3)	133 (97.1)	10.0	4 to 16
25–34	288 (94.7)	214 (95.5)	0.8	-3 to 5
35–44	227 (93.4)	192 (96.0)	2.8	-1 to 7
45–54	133 (91.7)	134 (97.8)	6.7	2 to 12
55–64	95 (90.5)	52 (96.3)	6.4	-1 to 14
65+	67 (90.5)	73 (98.6)	9.0	2 to 16
**Education**				
None	127 (86.4)	80 (88.9)	2.9	-6 to 11
Primary	488 (91.7)	410 (97.4)	6.2	3 to 9
Secondary	294 (94.8)	259 (97.7)	3.1	0 to 6
Tertiary	23 (100.0)	27 (100)	0.0	-
University	22 (100)	22 (95.7)	-4.3	-13 to 4
**Religion**				
Anglican	225 (91.5)	248 (98.8)	8.0	4 to 12
Roman Catholic	90 (96.8)	80 (97.6)	0.8	-4 to 6
Pentecostal	97 (94.2)	98 (95.1)	1.0	-5 to 7
Muslim	525 (91.6)	362 (95.3)	4.0	1 to 7
Other	17 (89.5)	10 (100)	11.7	-2 to 26
**Overall**	**954 (92.3)**	**798 (96.6)**	**4.6**	**3 to 7**

^α^Details of those that answered “No” and “Don’t know” are shown in S1 Table.

In some cases, respondents directly reported ADEs to CHEWs, upon experiencing an occurrence. They had a positive attitude towards reporting negative effects of a drug because they thought CHEWs would help and act on their report. For example, through changing their medication:

*“I tell them*, *when I use a drug and I see that am not improving*, *I inform the health worker and he changes treatment*.*” (FGD male*, *33 –Bukwaya)*

*“I went and reported to the basawo (doctors)about the medicine having burnt my skin and they changed my treatment*.*” (FGD female*, *28—Busowobu)*

On the other hand, some communities believed that CHEWs were too busy and had no time to attend to them. Community members trekked long distances from home to the health facility, spent the whole day there, and at the end of the day, drugs were sometimes out of stock. They felt CHEWs had not been helpful or responsive when reporting ADEs. Furthermore, some respondents felt that possible side effects of a drug were not explained by the CHEWs before prescribing. Some claimed that the medicines they bought from pharmacies did not produce the same reaction as those from government hospitals. Thus, some people resorted to going directly to private drug shops and clinics instead of wasting time at a health facility to only receive a referral.

*“The health worker gave me the drug and he did not tell me that it might make me feel that way*. *He told me to swallow the tablets and that is what I did*, *but when I bought it from the pharmacy*, *it did not give me the effects that I got from what I received from the government hospital*.*” (FGD female 36*, *Bukwaya)*

Others had not reported these events to CHEWs as they considered the effects to be a result of the drug’s strength:

*“…I did not report to anyone because I thought it’s because the drug was very strong*.*” (FGD male 37, Buwaiswa)*

### Respondent reporting of ADEs

The overall percentage difference in the number of respondents that reported having encountered or experienced an ADE was 115% (95% CI: 137% to 217%). However, there are variations among socio-demographic groups. For example, there was a reduction among those who had attained a tertiary education level compared to other education levels after the intervention. Additionally, respondents who identified as Roman Catholic reported the highest percentage increase compared to those of other Christian religions (Table 5).

**Table 5:** Comparison of respondents’ having ever experienced ADEs by socio-demographic characteristics before and after CDS^β^.

	Yes			
	Before (%)	After (%)	% relative diff.	95% CI
**Age group**				
15–24	51(31.3)	43(43.0)	37.4	18 to 57
25–34	109(35.9)	61(42.1)	17.3	2 to 32
35–44	80(32.9)	61(47.3)	43.8	28 to 60
45–54	47(32.4)	43(46.7)	44.1	24 to 64
55–64	36(34.3)	13(35.1)	2.3	-28 to 32
65+	26(35.1)	16(36.4)	3.7	-26 to 34
**Education**				
Primary	180(33.8)	120(42.4)	25.4	14 to 37
Secondary	109(35.2)	73(40.1)	13.9	0 to 28
Tertiary	12(52.2)	8(40.0)	-23.4	-68 to 21
University	10(45.5)	13(65.0)	42.9	16 to 70
**Religion**				
Anglican	89(36.2)	66(38.6)	6.6	-9 to 22
Roman Catholic	23(24.7)	20(41.7)	68.8	41 to 96
Pentecostal	45(43.7)	32(46.4)	6.2	-16 to 29
Muslim	189(33)	115(45.6)	38.2	27 to 49
Other	3(15.8)	4(57.1)	261.4	195 to 328
Overall	349 (20.5)	237(44)	114.6	137 to 217

^**β**^Details of those that answered “No” and “Don’t know” are shown in S2 Table

On further probing, some members elaborated on their experiences:

*“I witnessed a woman who was in ART clinic*. *When she took drugs*, *the whole body was burnt out with blisters*. *The patient immediately reported the problem and she claimed that since she started using drugs*, *she never experienced such*. *I think that was the drug effect*.*” (FGD male 40+*, *Busowobu)*

One respondent explained how her hand had become paralyzed due to a family planning injection she was given at the clinic. Some participants admitted to taking more of a drug than was prescribed:

*“I did not take the medicine as prescribed*.*” (FGD female 28*, *Busowobu)*

Another respondent reported an eye defect that she had witnessed in her relative.

*“[Relative] was given ARVs and Septrin tablets but got eye defect*. *The eye turned red and was not seeing properly*.*” (FGD female 35*, *Bukwaya)*

### Barriers to reporting

Respondents feared reporting ADEs and sympathized with the CHEWs who had issued the drug because they might lose their job as a result.

*“In case you report*, *health workers can lose their job which is not good because most of the times when you speak the truth*, *they ask you which doctor gave you the medication*.*” (FGD female 36*, *Bukwaya)*

Others reported that CHEWs were too harsh and therefore feared reporting to them for risk of being injured or harassed.

*“Some health workers are harsh and I fear to be injured*.*” (FGD male 40*, *Busowobu)*

*“If I am given an under dose and there is no improvement*, *when I report health workers might harass me*.*” (FGD female 28*, *Bukwaya)*

The other barrier to reporting these events was poverty. Some people who live far from the health facility may not have transport to take them to the facility:

*“Sometimes*, *people don’t have money to travel and take their complaints to the health facility*.*” (FGD female 45*, *Buwaiswa)*

Furthermore, buying cheap drugs from illegal drug outlets in rural areas reduced confidence to report any ADEs.

### Commonly reported ADEs

After CDS program, there was a significant increase in the population who would consider reporting serious reactions (19%, 95% CI:16% to21%), reactions to newly introduced drugs (15%, 95% CI:11to18%), unexpected reactions (16%, 95% CI: 13% to 19%), and reactions due to herbal and conventional medicines taken together (20%, 95% CI: 16 to 24%) (S3 Table).When respondents ranked potentially reportable ADEs, “serious reactions”were most commonly identified as being an ADE type compared to before the CDS program.

### Acceptability of the CDS program on reporting ADEs

Community members appreciated the role of community dialogues in creating awareness of drug use and the potential effects on member’s lives.

*“These meetings are good*. *It is through such meetings that community members can be aware of these effects and how to handle them*.*” (FGD female 28*, *Buwaiswa)*

The community members showed willingness to participate in future community dialogues to learn more about drugs and their proper use. They requested that meetings be extended to the village level to include those who may not have transport to health facilities. They recommended more mobilization of participants through churches, mosques and other health facilities.

*“Yes I would participate again because*, *I*, *for example*, *I did not know all that you have told us here*. *So*, *the health workers should be present as well*. *But please make it short next time*.*” (FGD female 39*, *Busowobu)*

*“I can participate again but you should consider holding these meeting in the communities as well like at the village level*. *Some people may not have transport to bring them at the health facility*.*” (FGD female 38*, *Bukwaya)*

Some community members felt that it would be important to engage schools to deliver these messages to children, as they also need to be aware of drug-related effects:

*“More to that*, *I think also the children need to know about these effects*. *I also suggest that you visit schools and talk to our children about these effects*.*” (FGD male 39*, *Bukwaya)*

Community members also appreciated the use of community dialogues as a tool to improve or create relationships between patients and CHEWs. They believe that once the dialogues are held together with CHEWs, it would be easier for patients to report ADEs.

*“…they help to build good relationship between patients and health workers*. *For example if we hold these meeting together with them*, *they cannot be harsh to us when we approach them*.*” (FGD male 33*, *Bukwaya)*

The community also mentioned that these dialogues would help members appreciate the role of concerned authorities, like the NDA:

*“These meetings will help our communities to appreciate the role of organizations like NDA through educative meetings like this*.*” (FGD male 42*, *Buwaiswa)*

### Recommendations for improving the CDS program for ADE reporting

#### A patient-friendly environment

Community members suggested that CHEWs should change their attitude towards patients and be open and polite to them. Thus, patients would be more willing to report any ADEs.

*“Health workers should be polite and free with patients because when he becomes free with his patients*, *it makes it easy to report*.*” (FGD male 39*, *Busowobu)*

Respondents also suggested that CHEWs should ask follow-up questions when patients return to the health facility to check if they had experienced any ADEs after taking the drug. This can give the patient a chance to interact with CHEWs:

*“We need to be asked when we come here because if the reaction was got some time back*, *you cannot tell it to the health worker*.*” (FGD female 38*, *Busowobu)*

Other respondents proposed a suggestion box at the health facility for reporting ADEs to enable patient reporting without revealing their identities to CHEWs:

*“I think suggestion box should be brought at the facility so that the health workers does not see who has reported or not*.*” (FGD female 27*, *Bukwaya)*

Finally, community members suggested that CHEWs should be part of these community dialogues as a way of changing their attitudes towards patients:

*“The health workers should be part of these meetings as well*. *If they are around*, *they can also change their attitude*.*” (FGD Female 36*, *Bukwaya)*

#### Practical modes of information delivery

Community members suggested that information on ADEs be designed and delivered in the form of dramas or skits, or played on videos. Information can include the process of taking medicine, developing ADEs, and then their reporting to respective channels. Members explained other programs that used storytelling techniques had better turnout to the meetings.

*“It can also be in form of drama activities*. *I mean if you can engage some people to play some skits about how these effects come up and how to report them*, *I think the whole process would be interesting*.*” (FGD female 32*, *Busowobu)*

*“There are some programs that have been coming to our communities especially for HIV and they have been playing skits and dramas to deliver the message to the members*. *I think you can also adopt the same technique since they are so interesting and members don’t leave before it is over*.*” (FGD female 46*, *Buwaiswa)*

*“Others actually play them on videos*. *They come with projectors and play in the community gatherings and after the video*, *they interact with us trying to explain what the video was about*.*” (FGD female 28*, *Bukwaya)*

#### More intense media engagement

In addition to community dialogues, community members also suggested the use of media in delivering information on medicine safety. They wanted more engaging radio programs as these would capture the audience’s attention and engage listeners through calling-in and texting. The community also requested more literature, such as leaflets, in their local language.

*“… it is good to air same information over the radios and televisions or some literature provided to community members in their local languages*.*” (FGD male 35*, *Buwaiswa)*

### Programmatic changes to community dialogues

#### Timing of dialogues

Community members suggested that there should be a day, and possibly a time, set for these dialogues to improve awareness of the sessions. The majority suggested Sunday afternoons around 4 PM or 5PM.

*“We can hold them on Sunday in the afternoon around 4pm or 5pm*.*” (FGD male*, *37 –Buwaiswa)*

*“Yes*. *But not every Sunday*. *You can choose like two Sundays in a month or every last Sunday of the month*.*” (FGD male 40*, *Bukwaya)*

#### Duration of dialogues

Respondents suggested that dialogues should be short and precise to keep members interested and attentive. A maximum of one hour was suggested to allow for the continuation of their daily programs afterwards.

*“It would be so appropriate to shorten these meetings to allow us do other things*, *otherwise people will start running out before the meeting is done*.*” (FGD male 35*, *Bukwaya)*

*“They should not take too long*. *It should be shortened to around 1 hour or two hours maximum*.*” (FGD female 42*, *Bukwaya)*

#### Frequency of dialogues

Community members appreciated the community dialogues and suggested routine sessions to stay informed.

*“You should always come here and hold these meetings with us so that we can know more about these effects and we might even forget and return to our old ways”*. *(FGD male 39*, *Buwaiswa)*

### Best way to engage the community

**Fig 1:**
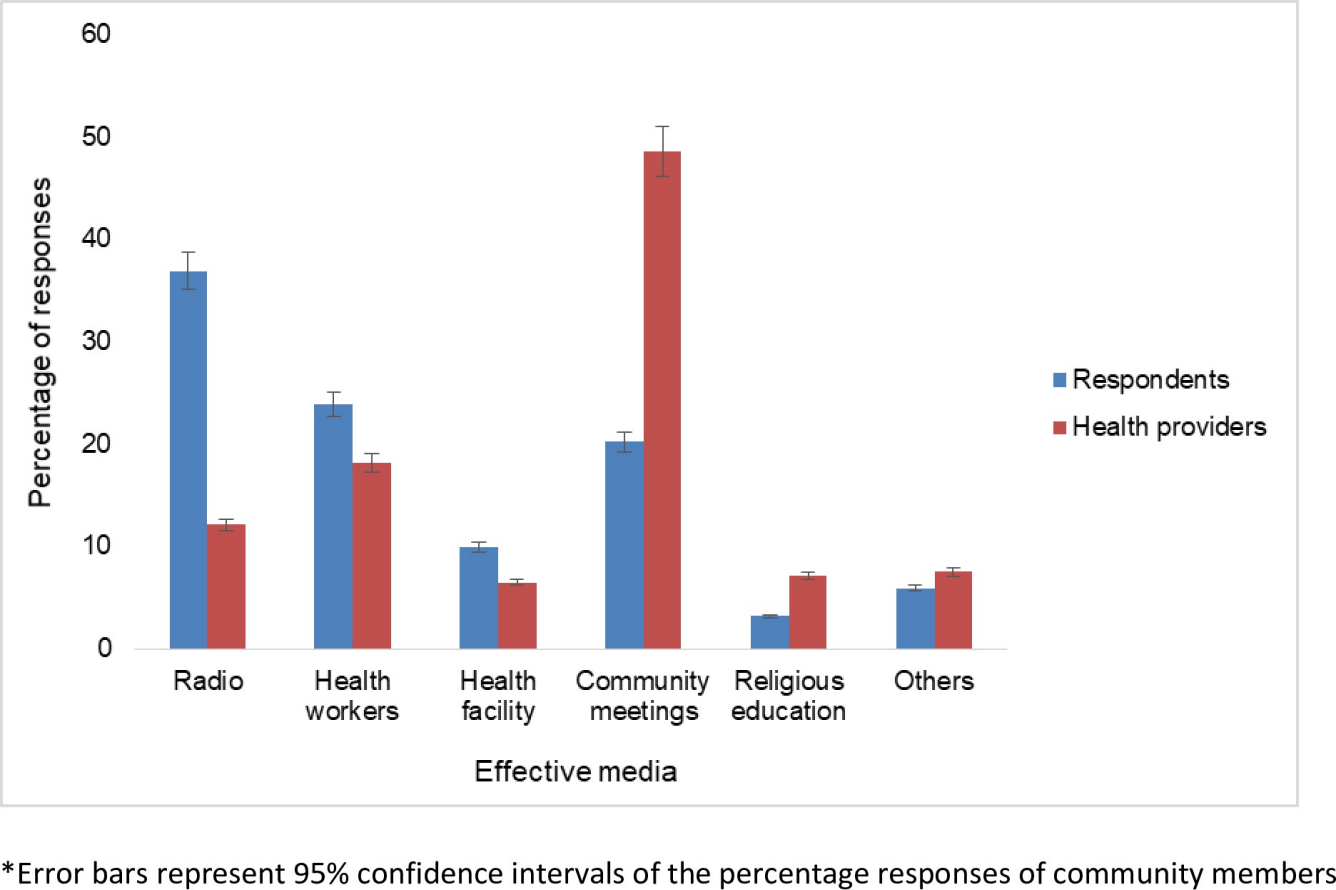
Comparison of options for reaching community members with ADE messages as perceived by respondents and healthcare professionals.

Among respondents, the radio was reported as the best way to deliver messages for sensitizing community members to ADE reporting. In contrast, health professionals regarded community meetings as the best method for reaching members (Fig 1). CHEWs and health facilities were considered to play an increasingly vital role in ADE sensitization, as perceived by both groups. Residents of the community were happy with the community dialogue meetings as a way of raising their awareness and as the best way to engage the community on ADEs (S1 Fig).

## Discussion

Community health and development work in resource-limited settings greatly benefits from program evaluations and programs developed to improve conditions in local communities. There is a paucity of data evaluating such programs in the published literature. This study systematically investigated the merit and significance of efforts to raise community awareness of drug safety issues. Results show that the CDS program succeeded in increasing knowledge and attitudes towards reporting ADEs in rural communities like other evaluation studies of similar health programs [17–19].

Overall, the knowledge of ADEs increased in the community after the program. The program facilitators of familiar faces sparked critical thinking and open dialogue among participants. The extent to which facilitated community meetings can influence healthcare quality lies in the social capital that they raise. These meetings provided an opportunity for networking and essential practical and emotional support, leading to the formulation of positive action plans and solidarity. Indeed, evaluation of a similar program in Mozambique also demonstrated an increase in community knowledge on a health program interventions, despite a lack of baseline data for comparison [20].

The respondent’s attitudes towards reporting ADEs improved slightly after the CDS program. Community members who believed healthcare professionals were helpful were more likely to report any ADEs after the intervention. Moreover, health system factors and patient expectations of the health sector determined attitudes towards the reporting of such events. Possibly, respondent’s positive attitudes towards the CDS program were borne out of the realization that an opportunity for discussing drug safety issues had been created. The dialogues provided community members with an opportunity for improved understanding and a more active involvement in their own treatment as part of health service delivery. However, in some rural communities where patients spend long hours at the government health facility and often find medications are out-of-stock, health-seeking behaviors are low and self-medicating is common [21]. In our study, some patients felt shy to report any ADEs to the health facility when the medication had been bought from elsewhere, especially from drug shops or clinics. Such factors contributed to a negative attitude to the CDS program. Great emphasis needs to be placed on patient education regarding drug safety issues, and a provision for direct patient reporting of such issues to the pharmacovigilance authorities, both locally and internationally to provide solutions to the community medicine safety issues. Our study was run and assessed over a short time which could have affected the magnitude of attitude changes in comparison to similar program evaluations that have taken longer [22].

Regarding the practice of reporting ADEs, the community members provided feedback on the best means of being informed of drug safety issues, as well as where they would feel most comfortable reporting them. After the program, there was a relative increase in those who would consider reporting different types of ADEs, such as serious reactions, reactions to newly introduced drugs, unexpected reactions, and those due to herbal and conventional medications taken together. Considering the examples of ADEs given, members demonstrated their willingness and capability to report safety issues should the CDS program be fully implemented. These findings point to an increased potential and intention of respondents to report, but may not necessarily translate into more reports following the program. Actual consumer reports reporting would have been a more accurate measure. Currently in Uganda, patients and consumers report ADEs, either indirectly through their healthcare professional, or directly through the newly established online reporting system [23]. The positive value of direct patient reporting has also been observed in well-resourced countries [24].

This study had some limitations. Firstly, there was potential recruitment bias associated with a representative cross-sectional survey as the households and in some cases individuals from the IMHDSS sampled for the study before the program may have been different from those interviewed after the program. While it is true that community respondents could have had similar baseline information within the routine IMHDSS data collection parameters, this may have differed in the case for information on ADEs since it is the first time the topic was introduced in the area. Secondly, the same questions were asked before and after the CDS program. Participants who were exposed to the questionnaire before and after the program could have caused response bias due to a tendency for participants under observation to give a response that they think is expected by the interviewer. Thirdly, by design of the study, we got a snapshot of the community’s views, however, the blend of quantitative and qualitative methods used enabled the study team to capture a holistic and contextual view of the CDS program on the reporting of ADEs and drug-related issues in rural communities. Forthly, changes in attitudes are a long-term measure, which the four-month program was too short to effectively assess. The four months of intervention coincided with the periodic data collection of IMHDSS which usually takes place biannually for three months. The intervention took a short period of four months due to logistical reasons. Classically, the practice of reporting ADEs is measured by the completion of such reports. However, in this study, there was no actual reporting done. Therefore, we measured a “change in willingness to report” by community members. While members were willing to accept the program once rolled out, information on the process of rolling-out could be different during the implementation phase. Overall, these results are generalizable to similar resource-limited settings with a homogeneous population.

The CDS program was widely accepted in the community. Community members benefited from the knowledge imparted about drug safety issues and appreciated the role of pharmacovigilance authorities in promoting drug safety campaigns. Our study confirms that with well-designed effective communication and a conducive environment, the community can quickly learn new concepts. Similar results were found during the assessment of educational programs to improve attitudes of healthcare professionals, and thus, ADE reporting rates [25, 26].

During focus group discussions, respondents pointed out that improvements in patient-healthcare professional relations were pivotal in making the program acceptable to the rural community. The community members proposed more intense media engagement for mobilization, a patient-friendly environment, and a higher program coverage at the village level during program roll out. They requested that the message be dramatized in the form of plays. Furthermore, members proposed adjustments to the timing, duration and frequency of the community dialogue meetings, based on similar experience from other program they had participate in [27]. An important finding from this study is that success of the CDS program cannot be separated from the performance and expectations of the healthcare system in general.

The adoption of the community dialogue toolkit made a number of assumptions. We assumed that the respondents knew the common diseases that affected the population across different ages, as well as the treatment received. Other assumptions were that the respondents knew the different points of care in the community, were familiar with commonly experienced ADEs, and knew the reporting channels for any medicine safety issues. However, the respondents proved not to have as much knowledge as was assumed.

The CDS program had its own limitations. Community members feared victimization for reporting any issues to the healthcare professionals who prescribed the medications in the first place. They also feared that these healthcare professionals who frequently attend to their health needs would lose their jobs. The presence of these underlying fears, coupled with the absence of a system for directly reporting ADEs to the authorities, poses challenges for monitoring drug safety. Moreover, there is limited ability of community members to differentiate between ADEs and the progression of a disease. The implication of this is that there should be more frequent interaction between the patient and their healthcare professional. If addressed, solutions to these issues could lead to increased knowledge of medicine safety in these rural communities.

A successful direct patient or consumer ADE reporting system should be accompanied by increased sensitization, delivered through multiple channels, such as radio messages, posters and brochures to raise drug-safety awareness, encourage dialogue, and involve the community in designing pertinent solutions. It should also utilize reporting tools that are tailored to the level of literacy and understanding of the rural community, while offering effective feedback those reporting about the ADE they have reported.

## Conclusion

In conclusion, the results of the current study showed that the CDS program increased knowledge and attitudes towards reporting ADEs. Despite having some prior knowledge that medicines have a potential to cause harm, the community showed signs of willingness to report the occurrence of ADEs through their healthcare professionals. This community expressed their readiness to accept the CDS program should it be rolled out and proposed adjustments for future implementation. In its current state, the CDS program was embraced as highly beneficial and could be adapted for direct patient or consumer reporting of ADEs in limited resource settings. A consideration for future research is the use of a control group in the evaluation of similar community programs, and the impact or long-term effects of the CDS program on the community. Further research is needed on direct patient ADE reporting systems compared to existing and indirect reporting through healthcare professionals, the cost effectiveness of various sensitization methods, and best practices for providing feedback to reporters in resource-limited settings.

## Supporting information

S1 File KAP study on adverse drug reactions–Household questionnaire. This was the questionnaire used to collect data about the households involved in the baseline and end-line surveys conducted before and after implementation of the community dialogues and sensitization.
(PDF)

S2 File Knowledge Attitudes and Practice (KAP) study on adverse drug reactions–Provider questionnaire. This questionnaire was used to collect data about the healthcare professionals in the community-based health facilities involved in the baseline and end-line surveys. At least two healthcare professionals from each health facility were randomly-selected.
(PDF)

S3 File FGD Interview Guide. **Focus Group Discussion Guide.** These set of questions guided the FGDs to probe for KAPs of the community and the attributes of CDS program for reporting adverse drug events that could either foster or hinder its uptake within communities.
(PDF)

S1 Table Comparison of study participants’ attitude towards the need to report adverse drug events before and after the CDS program. Comparison of study participants’ attitude towards the need to report adverse drug events before and after the CDS program including all responses to the question, “Is there a need to report adverse drug effects ?”
(PDF)

S2 Table Comparison of reporting potential CDS. **Comparison of respondents’ having ever experienced ADEs by socio-demographic characteristics before and after the CDS program.** A comparison of the potential to report adverse drug events in response to the question of whether participants had ever experienced one before and after.
(PDF)

S3 Table Commonly reported ADEs. A comparison of the type of adverse events that the respondents would report before and after the CDS program.
(PDF)

S1 Fig Best way to engage the community. Comparison of options for reaching community members with ADE messages as perceived by respondents and healthcare professionals.
(PDF)
