# Supplementary material for: The effect of community dialogues and sensitization on patient reporting of adverse events in rural Uganda: Uncontrolled before-after study
Source: PLoS One. 2019 May 9;14(5):e0203721. doi: 10.1371/journal.pone.0203721 (PMC6508596; doi:10.1371/journal.pone.0203721)
Supplement: S1 File — This was the questionnaire used to collect data about the households involved in the baseline and end-line surveys conducted before and after implementation of the community dialogues and sensitization. (PDF) [file pone.0203721.s001.pdf]

MAKERERE UNIVERSITY Center for Health & Population Research (MUCHAP) Iganga/Mayuge HDSS
KAP STUDY ON ADVERSE DRUG REACTIONS
RESPONDENT SHOULD BE ADULT (18 YRS +) USUAL RESIDENT OF THE HOUSEHOLD

IDENTIFICATION

FA CODE ☐☐☐ INTERVIEW DATE ☐☐☐☐☐☐☐☐

DISTRICT ……………………………………………………………..

VILLAGE NAME AND CODE ………………………………………………………………☐☐☐☐☐☐

LOCATION ID ☐☐☐☐☐☐☐☐☐☐☐☐

RESPONDENT NAME ………………… RESPID: ☐☐☐☐☐☐☐☐☐☐☐☐☐☐☐☐

HOUSEHOLD HEAD NAME ……….

HOUSEHOLD HEAD ID ☐☐☐☐☐☐☐☐☐☐☐☐☐☐☐☐

**Status of Visits by Interviewers:**	
*Result Codes<br>01 Completed<br>02 Respondent not at home at the time of visit<br>03 Respondent absent for extended period of time<br>04 Postponed	05 Refused<br>06 Others________________________<br>(Specify)

Editor **Code** ☐☐ **Date** ☐☐☐☐☐☐☐☐
Supervisor **Code** ☐☐ **Date** ☐☐☐☐☐☐☐☐
Data Entry **Code** ☐☐ **Date** ☐☐☐☐☐☐☐☐

QUESTIONS AND FILTERS	CODING CATEGORIES	SKIP
1. Wazalibwa mwaka ki, mwezi ki era lunaku ki?<br>*In what year, month and Day were you born?*<br><br>IF MONTH NOT KNOWN RECORD 99 & ALSO, IF YEAR IS NOT KNOWN RECORD 99	Years ☐☐☐☐<br>Months ☐☐<br>Day ☐☐	
2. Wali nemyaka emeka ku mazalibwa go agasemba yo?<br>*How old were you on your last birthday?*<br><br>COMPARE & RECONCILE Q1 AND Q2 IF NOT CONSISTENT	Age ☐☐	
3. Wasomaku?<br>*Have you ever attended school?*	Yes 1<br>No 2	→5
4. Kibiina ki ekyawaigulu kyewamaliliza?<br>*What is the highest grade you completed?*	1 – Primary<br>2 – Secondary Class ☐☐<br>3 – Tertiary<br>4 – University	
5. Obaire oviira mu maka gaano bulidho?<br>*Are you a usual resident of this household?*	Yes 1<br>No 2**(End)**	
6. Oli mufumbo?<br>*Are you currently married?*	Yes 1<br>No 2	
7. Oliwa eidhini ki?<br>What is your religion?	Christian(Anglican)…………………….1<br>Christian (Roman catholic)……..……2<br>Christian (Pentecostal)………………3<br>Muslim…………………………………4<br>Other (specify)…………………………5	
8. Omukulu wa maka gaano akweta atya?<br>What is your relationship with the Head of Household?	Head of Household………………… 01<br>Spouse………………………………… 02<br>Son/Daughter…………………………03<br>Step child……………………………. 04<br>Adopted/Foster child………….......05<br>Son in law/Daughter………………..06<br>Parent…………………………………07<br>Step-Parent………………………… 08<br>Parent-in-law……………………......09<br>Brother/sister……………………….....10<br>Brother-in-law/Sister-in-law………..11<br>No relation………………………......12<br>Other (specify)…………………………13	

9. Okola mulimo ki omukulu? (Omulimo ogusinga okutwala ebiseera byo buli lunaku)<br>What is your **main** occupation (ie. Activity that regularly takes most of your day time) **PLEASE CIRCLE ONLY ONE RESPONSE**	None………………………………...........1<br>Public sector employee (Professional)….. 2<br>Public sector employee (Manual)……......3<br>Private sector employee (Professional)… 4<br>Private sector employee (Manual) ……...5<br>Self-employed (business)…………….......6<br>Self-employed (Agric/fishery) ………...7<br>Domestic work in Household……..…… 8<br>Teacher……....…………………………… 9<br>Student…………………………………..10<br>Job-less……..……………………………..11<br>Incapacitated………………………........12<br>Other(specify)……………..……………..13	
**SECTION 2: OBULWAIRE OBUMANHIKWA MU MAKA**<br>**COMMON ILLNESSES IN THE HOUSEHOLD**		

10. Buti, nandyenze okukubuzaku ku'ndwaire eghe oba abantu bo mumaka gaano dhebaalwalaku mu myezi esatu egibise n'obwiidandhabi bwemwafunaku okudidandhaba. Nkusaba onkobereku amainha ag'omuntu, emyaka, obulwaire n'obwiidandhabi obwa mughebwa.

Now, I would like to ask you about the illnesses that you or members of your household have experienced in the last 3 months and the treatment you received to manage them. I request you to tell me the name of the person, age, illness and drugs/treatment taken.

Name	AGE	ILLNESS	TREATMENT TAKEN	
i).				
ii).				
iii).				
iv).				
v)..				
vi).				

**11a. Ighe oba abantu b'omumaka gaano mufunawa obwiidandhabi bwemukozesa?**<br>Where do you or members of your household usually get the treatment you use from? **(CIRCLE ALL APPLICABLE)**	Drug shop ……………………………1<br>VHT…………………………………….2<br>Private clinic ………….....…………3<br>Government HC……………………… 4<br>Hospital………………………………5<br>Relative/Neighbour …...……………6<br>Other patients……………………….7<br>Herbalist ………………………………8<br>Friends……………………………….9<br>Other shops/Market ………………10<br>Bus………………………………………11<br>Other, Specify……………………...…12	
**11b. Abasawo bakuwaku amawulire gaano wamanga agagema kubulezi nga ogiire okufunha obwiidandhabi?**<br>**Do health care providers give you the following information about the medicines when you seek treatment**	Drug name.... 1. Yes 2. No 3. DKN<br>What it treats 1. Yes 2. No 3. DKN<br>How to take it 1. Yes 2. No 3. DKN	

SECTION 3: AMAWULIRE AGAGEMA KU BULEZI, OBUZIBU N'OKULOOPA

KNOWLEDGE ABOUT DRUGS, SIDE EFFECTS AND REPORTING

12. Obaire okiidhi nti mukwongereza kukwiidandhaba obulwaire oba endwaire, obulezi bwonabwona busobola okuleeta obuzibu ku muntu abukozeyisa?<br><br>Are you aware that in addition to treating illness/diseases, any medicine has potential to cause negative effect to person taking it?	Yes ……………………………………1<br>No ……………………………………2<br>Don't know ……………………………3	
13.Mu myezi esatu egibise, wawulilaku obubaka bwonabwona obugemagana nobulabe oba obuzibu obulezi bwebusobola okututuusaku nengeri y'okuloopa obuzibu obwo?<br>In last 3 months, have you heard any information about the possible negative effects of the medicines/drugs that we take and how to report these effects?	Yes ………………………………………<br>No ………………………………………	
14.Wawulirawa obubaka obugemagana kubulabe obuva ku bulezi mu myezi 3 egibise?<br>Where did you hear information about the drug /medicine effects in last 3 months?<br><br>**(MULTIPLE RESPONSE ALLOWED)**	Radio………………………………………1<br>Health worker told me……………………2<br>Discussion at Health facility………………3<br>Discussion in the community……………4<br>Poster/publications……………………5<br>Other(Specify)…………………………6	
15.Okukubagania ebidhubo nokusomesa abantu munkiiko kubulabe obututusiibwaku okuva ku obulezi n'okuloopa obulabe obwo, olowooza nganenkola enayongera mubantu okuloopa obulabe obuva mubulezi?<br>Do you think conducting community meetings to discuss and sensitize people about possible drug effects and reporting is an effective way to increase reporting of drugs reactions?	Yes ………………………………………<br>No ………………………………………	
16.Okukubagania ebidhubo nokusomesa abalwaire mu malwaliro kubulabe obututusiibwaku okuva ku obulezi n'okuloopa obulabe obwo, olowooza nganenkola enayongera mubantu okuloopa obulabe obuva mubulezi?<br>Do you think conducting health facility meetings with patients to discuss and sensitize them about possible drug effects and reporting is an effective way to increase reporting of adverse drug reactions?	Yes ………………………………………<br>No ………………………………………	
17.Mu ndowoozayo, **ngeriki esingirailala obulungi mukwongera** okuloopa obulabe obututusibwaku nga buva kubulezi?<br>**(DON'T READ OUT OPTIONS)**<br>In your opinion, what is the **most effective** way to increase reporting of effects the drugs/medicines that we take in this community?	Radio……………………………………1<br>Health education by health worker … 2<br>Discussion at Health facility…………3<br>Discussion in community meetings….4<br>Education by religious leaders ………5<br>Other, specify…………………………6	
18.Wa ensonga lwaki olowooza nga nesinga?<br>Give reasons why you think it's the best way?	………………………………………<br>………………………………………<br>………………………………………	
19.Ngeliki edhindi dholowoza nga nedhandi kozeseibwa mu kumanisa abantu kubulabe obuva kubulezi nho kuloopa?<br>Which other ways would you suggest to be used in informing community members about ADRs and reporting?	Way 1.:……………………………<br>Way 2:……………………………<br>Way 3:……………………………	
20. Eghe oba omuntu w'omumaka gaano wafunaku obuzibu bwonabwona obuva ku bulezi?<br><br>Have you or a member of your household ever experienced any negative effect of the medicines	Yes ……………………1<br>No ……………………2 **(Skip to q22)**<br>Don't know… …………3 **(Skip to q22)**	

21. Wakolaku ki oba omuntu w'omumaka gaano yakola ki nga afunye obuzibu obwo?<br><br>What did you or member of your household do in the event that you/other member experienced that negative effect?	Report it back to provider……………........1<br>Treat it myself……………..........…...........2<br>Ignored it…………………….………….........3<br>I stopped the drug.......................................4<br>Don't know………………………………..........5	
22. Osobola okunkoberaku nti obulumi bwowulira nga omazze okufunha obwiidandhabi buva oba bwali buva ku bulezi bwewakozesa?<br>Would you tell if the unwellness or suffering you feel after treatment is/was as a result of the medicine you took?	Yes ………………………………………........1<br>No……………………………………………......2<br>Dont know...3	
23a. Wandilopye?<br><br>Would you report such?	Yes……………………………………….........1<br>No………………………………… 2(**SKIP TO Q24**)<br>Don't know……….....………………………… 3	
23 b. Waloopa?<br><br>Did you report the Adverse Drug Reactions:	Yes………………………….........1**Skip to q25a)**<br>No….....………………………… 2<br>Don't know………....……...… 3	
24. Oba mbe, lwaki ti waloopa buzibu buno obw'obulezi?<br><br>If No, why wouldn't you report the negative effects of the medicines?	Cannot tell if due to drug or not… ....1<br>Health workers have no time………….........2<br>I fear being victimised................................3<br>It will disappear shortly.............................4<br>It is not necessary.….………………….........5<br>Other, specify…………………………..........6	Skip to Q26
25a. Okuva ebiseera byewazuura obuzibu, wamala eibanga ki okuloopa obuzibu bunno?<br>From time of detection of the Adverse reaction, how long did you take to report that adverse Event?	……………days or<br>…………..weeks or<br>…………… Months<br>……………..N/A (CHOOSE Appropriately)	
25b. Oba waloopa, kyikyi ekyakolebwawo?<br><br>If you reported what happened?	Counselled about the effect of drug.........1<br>Retreated...2<br>Drug was changed......................................3<br>Told to continue taking the drug..............4<br>Treatments was dicontinued ....5<br>I was ignored..6<br>Clinician was busy treating others… ....7<br>Other, specify……………………………..........8	Q27
26. Singa wali wa kuloopa, wandi loopye gha?<br>If you were to report where would you report it to?	To the nearest health facility.....……….......1<br>Report to where drug was got from.........2<br>VHT…………………………………………......3<br>District Health Office (DHO)......................4<br>Local authorities (S/County health worker & LCs..5<br>National Drug Authority (NDA).................6<br>Other, specify……………………………..........7	
27a. Okusinziira kwiighe, ngeri ki dhobona nga nesinga era nga nungi ku kuloopa embera embi mu maka gaano?<br>According to you, what method, would you prefer as the **best** and **convenient** system for reporting an adverse event in this household?	………………………………………………..	
27b. Okusinziira kwiighe, ngeri ki kudhino wamanga doboona nga nedisinga era nga nungi ku kuloopa embera embi mu maka gaano?<br>According to you, which of the following methods, would you prefer as the **best** and **convenient** system for reporting an adverse event in this household? (**SELECT ONLY ONE**)	Phone call to the clinic………………….......1<br>Phone call to NDA......................................2<br>Phone call to DHO......................................3<br>SMS…………………………………………........4<br>Returning to the drug seller……………......5<br>Report to VHT……………………………........5<br>Local authority(S/County health worker & LCs)..6<br>Other, specify……………………………..........7	

28. Olowooza okuloopa obuzibu obuva mu kukozesa obulezi kyetagisa?<br>Do you think reporting adverse drug reaction is necessary?	Yes……………………………………………………1<br>No……………………………………………………2<br>Dont know…………………………………………3	

Section 4

Mu kitundu kino, nkusaba nti owulilize ku bintu bino byenja okukusomera, era nsaba okunkobera nti yii, mbe oba tiidhi

In this section, I request that you listen to the statement I am going to read to you, and please tell me you "Agree"/"YES" or "Disagree"/"No" OR "Don't-know"(Tick in the appropriate box provided)

Bino bili n'okuloopebwa?<br>*These should be reported?*		*Yes/Agree*	*No/Disagree*	*Don't Know*
1.	Obuzibu obulowozebwa okuva ku bulezi obutamanikwa<br>Suspected negative effects for which suspected drugs is uncertain			
2.	Obukakafu okuva ku bulezi oba obuzibu<br>Certain/sure reactions or negative effects			
3.	Obuzibu obw'amanhi einho nga okuvaku enviiri, nebindhi okuva mubwiidandhabi bwa kokolo<br>Serious reactions e.g. sight loss due to quinine, hair loss from cancer drugs etc			
4.	Obuzibu nga butono nga okusesema, okwiidhukana<br>Mild reactions e.g. side effects such as vomiting & diarrhea			
5.	Obuzibu nga obufunye okuva mu bulezi obubaire butundibwa mu butale okuswiika emyaka eikumi<br>Reactions to drugs which have been on sale (in the market) for more than 10 years			
6.	Obuzibu okuva ku bulezi obuyaka obufulumizibwa mu butale<br>Reactions to newly introduced drugs in the market			
7.	Obuzibu obumanhikwa obulungi<br>Common or well known reactions			
8.	Obuzibu obutasubirwa oba obutali bwa bulidho<br>Unexpected/Unusual reactions			
9.	Okusobola okugemaganhia n'obulezi obundhi<br>Possible interaction with other drugs			
10.	Obuzibu obuva mu kukozesa eidhagala lyekinaasi<br>Reactions that may result from taking herbal medicine			
11.	Obuzibu obuva mu kukozesa eidhagala lyekinaasi ghalala n'obulezi obwekizungu okugeza nga mukiseera kya mabundha, akakowoolo, omusudha n'ebindhi.<br>Reactions from taking herbal and modern medicine together eg. During pregnancy, for cough, fever etc			

Ndowooza ki dhowa kukuloopa obuzibu bw'ofuna nga omazze okukozesa obulezi era dhandisanile dhiloopebwe dhitya?

What are your comments about reporting adverse drug reactions and how they should be reported.

………………………………………………………………………………………………
………………………………………………………………………………………………
………………………………………………………………………………………………
