# Supplementary material for: The effect of community dialogues and sensitization on patient reporting of adverse events in rural Uganda: Uncontrolled before-after study
Source: PLoS One. 2019 May 9;14(5):e0203721. doi: 10.1371/journal.pone.0203721 (PMC6508596; doi:10.1371/journal.pone.0203721)
Supplement: S2 File — This questionnaire was used to collect data about the healthcare professionals in the community-based health facilities involved in the baseline and end-line surveys. At least two healthcare professionals from each health facility were randomly-selected. (PDF) [file pone.0203721.s002.pdf]

MAKERERE UNIVERSITY CENTER FOR HEALTH & POPULATION RESEARCH (MUCHAP) - Iganga/Mayuge HDSS
National Pharmacovigilance Centre (NPC), National Drug Authority (NDA)

KAP STUDY ON ADVERSE DRUG REACTIONS - PROVIDER QUESTIONNAIRE

Questionnaire-No……..
FA-Name: …………………………………………………………………...................FA CODE:………….
Respondent's identification information

Date	Respondent Surname: ……... ....<br>Other Names: ………………………
Name of the facility	Age
FIN:	Gender: 1 =Male 2= Female
Village	Parish
District	**Cadre/Qualification**
	1. Doctor/Medical officer
	2. Clinical officer
	3. Nurse/Midwife
	4. Nursing Assistant
	5. Pharmacist
	6. Lab technician/ Assistant
	7. Counsellor
	8. Other (Specify)
	**Maximum education level attained**
	1. Masters
	2. Bachelors
	3. Diploma
	4. Certificate
	5. Secondary
	6. Primary
	7. None

Eidwaliro lino liri mu eidhala ki?<br>What is the facility classification	Hospital……………………………………………………………1<br>Health Centre IV………………………………………………………2<br>Health Centre III………………………………………………………3<br>Health Centre II…………………………………………………………4<br>Private Clinic……………………………………………………………5<br>Drug Shop…………………………………………………………………6<br>Pharmacy…………………………………………………………………6<br>Shop………………………………………………………………………7<br>Other (Specify)…………………………………………………………8
Eidwaliro lino litwalibwa anhi?<br>What is the ownership of this facility	Government owned……………………………………….1<br>NGO/Faith-based………………………………………..2<br>Private……………………………………………………………3

Section A

1.	Olowooza abantu b'omukitundu munno bateera kufunha gha obulezi?<br>Where do you think community members commonly get their medicines from?<br>**(MULTIPLE ANSWERS ALLOWED)**	Drug shop ……………………………………………………………1<br>VHT ……………………………………………………………………2<br>Private clinic ………………………………………………………3<br>Government clinic……………………………………………………4<br>Relative ………………………………………………………………5<br>Other patients ………………………………………………………6<br>Herbalist ……………………………………………………………7<br>Friends ………………………………………………………………8<br>Internet………………………………………………………………9<br>Market ………………………………………………………………10<br>Bus …………………………………………………………………11<br>Shops ………………………………………………………………12<br>Other, Specify………………………………………………………13
2.	Obaire okidi nti obulezi bwonabwona busobola okuba n'obuzibu/obulabe ku mulwaire?<br>Are you aware that any medicine may have negative effects on the patient?	Yes ……………………………………………………………………1<br>No………………………………………………………………………2<br>Don't know …………………………………………………………3

3.	Mu myezi esatu egibise, wawulilaku obubaka bwonabwona obugemagana nobulabe oba obuzibu obulezi bwebusobola okututuusaku nengeri y'okuloopa obuzibu obwo?<br>In last 3 months, have you heard any information about the possible negative effects of the medicines/drugs that we take and how to report these effects?	Yes ....1<br>No...2
4.	Wawulirawa obubaka obugemagana kubulabe obuva ku bulezi mu myezi 3 egibise?<br>Where did you hear information about the drug /medicine effects in last 3 months?<br><br>**(MULTIPLE RESPONSE ALLOWED)**	Radio…………………………………………………………………….1<br>Health worker told me……………………………………………………..2<br>Discussion at Health facility………………………………………………3<br>Discussion in the community……………………………………………..4<br>Poster/publications…………………………………………………………5<br>Other(Specify)………………………………………………………………….6
5.	Okukubagania ebidhubo nokusomesa abantu munkiiko kubulabe obututusiibwaku okuva ku obulezi n'okuloopa obulabe obwo, olowooza nganenkola enayongera mubantu okuloopa obulabe obuva mubulezi?<br>Do you think conducting community meetings to discuss and sensitize people about possible drug effects and reporting is an effective way to increase reporting of drugs reactions?	Yes ....1<br>No...2
6.	Okukubagania ebidhubo nokusomesa abalwaire mu malwaliro kubulabe obututusiibwaku okuva ku obulezi n'okuloopa obulabe obwo, olowooza nganenkola enayongera mubantu okuloopa	Yes ....1<br>No...2

	obulabe obuva mubulezi?<br>Do you think conducting health facility meetings with patients to discuss and sensitize them about possible drug effects and reporting is an effective way to increase reporting of adverse drug reactions?	
7.	Mu ndowoozayo, **ngeriki esingirailala obulungi mukwongera** okuloopa obulabe obututusibwaku nga buva kubulezi?<br>**(DON'T READ OUT OPTIONS)**<br>In your opinion, what is the **most effective** way to increase reporting the effects of drugs/medicines that we take in this community?	Radio………………………………………………...……………….1<br>Health education by health worker ……………………………….2<br>Discussion at Health facility………………………………..………3<br>Discussion in community meetings……………………...……4<br>Education by religious leaders ……………………………………5<br>Other, specify..
8.	Wa ensonga lwaki olowooza nga nesinga?<br>Give reasons why you think it's the best way?	………………………………………………………………….<br>…………………………………………………………………...<br>……………………………………………………………………
9.	Ngeliki edhindi dholowoza nga nedhandi kozeseibwa mu kumanisa abantu kubulabe obuva kubulezi nho kuloopa?<br>Which other ways would you suggest to be used in informing community members about ADRs and reporting?	Way 1.:...<br>Way 2:...<br>Way 3:..
**10.**	Magezi ki g'owa omulwaire okukola singa aba afunye obuzibu?<br>What would you advise a patient to do in case they experienced a negative effect?<br>(MULTIPLE ANSWERS ALLOWED)	Report it to a health worker..1<br>Treat it themselves...2<br>Ignore it/do nothing...3<br>Stop the medicine...4<br>Report to NDA/DHO...5<br>Don't know ....6<br>Other (specify)...7
11.	Oba ti kuloopa, lwaki?<br>If not reporting, why?	Cannot tell if ADR or not 1

		I have no time ....2<br>The fear of being sued or victimised ....3<br>It will disappear shortly ....4<br>It is not necessary ....5<br>Don't know..6<br>Other, specify 7
12.	Singa obaire wa kuloopa obuzibu obwo obuva ku bulezi, wandibaire oloopa gha?<br>If you are to report the negative drug effect or ADR, where would they/you report it to?	District Health Office (DHO)..1<br>District Drug Inspector (DDI) ....2<br>Send to nearest health facility ....3<br>Send to NDA regional office..4<br>Call the clinic where drug was purchased ....5<br>VHT ....6<br>Local authorities (Sub County health workers & LCs)..7<br>Other, specify..8
13a.	Okusinziira kwiighe, ngeri ki dhobona nga nesinga obulungi okuloopa obuzibu obuva ku bulezi; **nga buva ku mulwaire okujja eri omwiidandhabi?**<br>According to you, which method would you prefer as the best system for reporting an adverse event from patient to healthcare provider?	....
13b.	Okusinziira kwiighe, ngeri ki kudhino wamanga doboona nga nedisinga obulungi okuloopa obuzibu obuva ku bulezi; **nga kuva ku mulwaire okujja eri omwiidandhabi?**<br>According to you, which of the following methods would you prefer as the best system for	Phone call to provider..1<br>SMS..2<br>Report to the drug seller..3<br>Report to PV coordinator at hospital ....4<br>Report by email..5<br>Report using mobile app..6<br>VHT 7

	reporting an adverse effect from patient to healthcare provider?<br>**(READ OPTIONS ALOUD)**	Local authorities ....8<br>Other, specify 9
14a	Okusinziira kwiighe, ngeri ki dhobona nga nesinga obulungi okuloopa obuzibu obuva ku bulezi **nga kuva eri omwiidandhabi oweby'obulamu okutubutuusa waigulu?** From healthcare provider onwards	…………………………………………………………………….
14b.	Okusinziira kwiighe, ngeri ki kudhino wamanga doboona nga nedisinga obulungi okuloopa obuzibu obuva ku bulezi nga **kuva eri omwiidandhabi oweby'obulamu okutubutuusa waigulu? From healthcare provider onwards**<br>**(READ OPTIONS ALOUD)**	Phone call to NDA...1<br>SMS to NDA ....2<br>Report to the drug seller..3<br>Report to PV coordinator at hospital ....4<br>Report by email...5<br>Report using mobile app..6<br>Local authorities ....7<br>Other, specify 8
15a.<br><br>15b	Nga omwiidandhabi, waloopaku obuzibu omulwaire wo bweyafuna ng'omaze okumuwa obwiidandhabi?<br>As a provider, have you ever reported an adverse event after treatment?<br>Waloopa gha?<br>Where did you report?	Yes ....1<br>No……………………………………………………2 **(SKIP to Q17)**<br><br>..
16	Waloopaku mubwangu ki?<br>How soon did you report that Event?	1 ………….days<br>2 …………weeks<br>3…………months<br>4………..N/A (Choose Appropriate)
17.	Olowooza okuloopa obuzibu	Yes 1

	obuva ku bulezi nga kyetagisa?<br>Do you think reporting adverse drug reaction is necessary?	No...2
18.	Omwiidandhabi weby'obulamu asobola okuloopa obuzibu obuva ku bulezi mukitundu kino?<br>Can healthcare provider report ADR in this community?	Yes ....1<br>No...2
19.	Omulwaire asobola okuloopa obuzibu obuva ku bulezi mukitundu kino?<br>Can a patient report ADR in this community?	Yes ....1<br>No...2
20.	Ofunaku amawulire ku bulezi n'obuzibu obuva mu bulezi bulidho?<br>Do you obtain information on medicines and their effects regularly?	Yes ....1<br>No.....................................2**(Skip to Q22)**
21.	Okuva gha?<br>From what source?	Online- internet ....1<br>National Drug Authority and other government agencies.................2<br>Non Governmental Organisations (NGOs).......................................3<br>Friends and relatives...4<br>Leaflets in medicine package ....5<br>Radio announcements...6<br>Newspapers ....5<br>Advertisements...6<br>Markets...7<br>Clinicians and drug sellers 8

	Posters at health facilities...9<br><br>Other, specify 10

Section B

Buti ndhikusaba okwiiramu nti yii, mbe oba tiidhiku bibuuzo bino;

*Now I would like to request you to respond either **Yes, No or Don't know** to the following questions.*
Bintu ki kubiino by'olowooza nti bili n'okuloopebwa?
*Which of these do you think should be reported? (**Tick** in the box provided)*

Bintu ki kubiino by'olowooza nti bili n'okuloopebwa?<br>*Which of these do you think should be reported?*	*Yes=1*	*No=2*	*Don't Know=99*
22. Obuzibu obulowozebwa okuva ku bulezi obutamanikwa<br>Suspected effect (for which suspected drugs is uncertain)			
23. Obuzibu okuva ku bulezi obumanikwa/obukakasibwa<br>Effects or reactions that are Certain/sure			
24. Obuzibu obw'amanhi einho nga buyinza okuviraaku okufa, okwegharikiriza obulamu, bwetagisa okugha omulwaire ekitanda oba buleteera okuba mu eidwaliro okumala eibanga eiwavu, ekiviraaku okweyongera obutesobola, kyaleteera okuzaala omwana ali n'obukosefu, oba kyetagisa okuyambibwa okutangira obukosefu obutali bwankalakalira<br><br>Serious reactions e.g. (***Results in death, life-threatening, requires inpatient hospitalization or causes prolongation of existing hospitalization, results in persistent or significant disability/incapacity, is a congenital anomaly/birth defect, or requires intervention to prevent permanent impairment or damage***)			
25. Obuzibu nga butono okuva ku bulezi okugeza nga omutwe okulumilira, okusesema n'okwiidhukana<br><br>Mild reactions e.g. side effects such as headache, vomiting & diarrhoea			
26. Obuzibu nga obufunye okuva mu bulezi obubaire butundibwa mu butale okuswiika emyaka eikumi<br><br>Reactions to drugs which have been on sale (in the market) for more than 10 years			
27. Obuzibu okuva ku bulezi obuyaka obufulumizibwa mu butale<br><br>Reactions to newly introduced drugs in the market			

28.	Obuzibu obumanhikwa obulungi<br><br>Common or well known reactions			
29.	Obuzibu obutasubirwa oba obutali bwa bulidho<br><br>Unexpected/Unusual reactions			
30.	Okusobola okugemaganhia n'obulezi obundhi<br><br>Possible interaction with other drugs			

Section C

	Buti ndhikusaba okwiiramu nti yii, mbe oba tiidhi ku nsonga dhenja okusomera edhiyinza okukulemesa okuloopa obuzibu obulowoozebwa okuva mu kukozesa obulezi<br><br>*Now I request you to respond YES, No or DK to each of the factors im going to read to you that may discourage you from reporting a suspected Adverse Drug Reaction or negative effects.*	*Yes=1*	*No=2*	*Don't Know=99*
31.	Nali tiidhi nti obuzibu obuva mu kukozesa obulezi buli n'okuloopebwa<br>I didn't know ADRs should be reported			
32.	Tyekakasa kyika kya buzibu obw'okuloopebwa<br>I am not sure of the type of reactions to be reported			
33.	Obutamanhisibwa bikka eby'okuloopa<br>Lack of knowledge of the forms for reporting			
34.	Empapula nzibu dakwiidhuza<br>Complex to fill the forms			
35.	Obutekakasa kubuzibu obuva ku bulezi obumilibwa<br>Uncertainty about reaction being due to drugs ingested			
36.	Okutya okuvunhanizibwa mu mbuga dha mateeka<br>Fear of a consequent law suit			
37.	Obutasasulwa nga oloopye<br>Non-remuneration for reporting			
38.	Obutaba nabiseera bya kuloopa buzibu obuva mu kukozesa obulezi<br>Lack of time to report ADR			
39.	Ensonga endhala etaloopebwa teyinza kukosa eiterekero ly'amawulire ag'obuzibu obuva mu kukozesa obulezi<br>A single unreported case may not affect ADR database			
40.	Obukalubiriivu okusalawo oba obuzibu okuva mu kukozesa obulezi			

bubairewo oba mbe<br>Difficult to decide whether ADR has occurred or not			

Gabanaku niife kundhowooza dho edhiyinza okutumbula mu kuloopa obuzibu obuva mu kukozesa obulezi
Share with us some suggestions on the possible ways to improve the ADR reporting.

………

………

………

………

………
