# Supplementary material for: The effect of community dialogues and sensitization on patient reporting of adverse events in rural Uganda: Uncontrolled before-after study
Source: PLoS One. 2019 May 9;14(5):e0203721. doi: 10.1371/journal.pone.0203721 (PMC6508596; doi:10.1371/journal.pone.0203721)
Supplement: S3 File — Focus Group Discussion Guide. These set of questions guided the FGDs to probe for KAPs of the community and the attributes of CDS program for reporting adverse drug events that could either foster or hinder its uptake within communities. (PDF) [file pone.0203721.s003.pdf]

FOCUS GROUP DISCUSSION INTERVIEW GUIDE

INTRODUCTION SCRIPT: *To be read by researcher/group facilitator*

You have been invited to take part in this research study which aims to assess community knowledge, practices and attitudes towards reporting of adverse drug events (ADEs). We also want to assess how best these ADEs can be identified, reported and acted upon.

For this discussion group, I will invite you to share as much as you feel comfortable sharing with the other patients and community members. This conversation will be recorded for only the researchers to hear. Any recording of you taken today will not be used without your express consent. You may choose not to speak, or may leave the discussion at any time, for any reason, without explanation. Your information will be treated with ultimate anonymity and confidentiality. Thanks.

QUESTIONS FOR DISCUSSION

1. Please tell us some experiences of you or any household member who has ever experienced any of this negative effect?
2. How could you tell if un-wellness you or other household member is feeling or suffering from is as a result of the medicine you/he/she took?
3. What would you do if you or a member of your household experiences that negative effect?
4. In this community where does one go to report any ADEs experienced? What are the available means of reporting these ADEs? Have you ever reported an adverse event?
5. Why do you think reporting adverse drug events is necessary?
6. In this community, what may be the reasons/factors for some people not reporting ADEs?
7. In your view, how can the reporting of these ADEs be improved so that we see increased reporting of ADEs in this community?
8. Tell me what you thought about participating in the Community Dialogues or meetings like this one
9. So what is your perceived appropriateness of having community dialogues?
10. What are your views on the potential effect these meetings can have on ADE reporting?
11. So how would you like to see these community dialogues occur in the future?
12. Would you participate in these again? Please tell me why or why not.
