# Supplementary material for: The effect of community dialogues and sensitization on patient reporting of adverse events in rural Uganda: Uncontrolled before-after study
Source: PLoS One. 2019 May 9;14(5):e0203721. doi: 10.1371/journal.pone.0203721 (PMC6508596; doi:10.1371/journal.pone.0203721)
Supplement: S1 Table — Comparison of study participants’ attitude towards the need to report adverse drug events before and after the CDS program including all responses to the question, “Is there a need to report adverse drug effects ?” (PDF) [file pone.0203721.s004.pdf]

Comparison of study participants' attitude towards the need to report adverse drug events before and after the CDS intervention.

	Yes				No				Don't know			
	**Before (%)**	**After (%)**	**% diff.**	**95% CI**	**Before (%)**	**After (%)**	**% diff.**	**95% CI**	**Before (%)**	**After (%)**	**% diff.**	**95% CI**
**Age category**												
15-24	144 (88.3)	133 (97.1)	10.0	4 to 16	12 (7.4)	4 (2.9)	-60.8	-88 to -33	7 (4.3)	0 (0)	-100.0	0
25-34	288 (94.7)	214 (95.5)	0.8	-3 to 5	8 (2.6)	8 (3.6)	38.5	21 to 55	8 (2.6)	2 (0.9)	-65.4	-88 to -42
35-44	227 (93.4)	192 (96.0)	2.8	-1 to 7	10 (4.1)	7 (3.5)	-14.6	-33 to 4	6 (2.5)	1 (0.5)	-80.0	-111 to -49
45-54	133 (91.7)	134 (97.8)	6.7	2 to 12	7 (4.8)	1 (0.7)	-85.4	-128 to -43	5 (3.4)	2 (1.5)	-55.9	-83 to -29
55-64	95 (90.5)	52 (96.3)	6.4	-1 to 14	4 (3.8)	1 (1.9)	-50.0	-90 to -10	6 (5.7)	1 (1.9)	-66.7	-113 to -20
65+	67 (90.5)	73 (98.6)	9.0	2 to 16	5 (6.8)	0 (0.0)	-100.0	-	2 (2.7)	1 (1.4)	-48.2	-
**Education**						0 (0.0)						
None	127 (86.4)	80 (88.9)	2.9	-6 to 11	12 (8.2)	7 (7.8)	-4.9	-30 to 20	8 (5.4)	3 (3.3)	-38.9	-67 to -10
Primary	488 (91.7)	410 (97.4)	6.2	3 to 9	22 (4.1)	7 (1.7)	-58.5	-74 to -43	22(4.1)	4 (1.0)	-75.6	-95 to -56
Secondary	294 (94.8)	259 (97.7)	3.1	0 to 6	12 (3.9)	6 (2.3)	-41.0	-59 to 0	4 (1.3)	0 (0.0)	-100.0	0
Tertiary	23 (100.0)	27 (100)	0.0	-	0 (0.0)	0 (0.0)	0.0	-	0 (0.0)	0 (0.0)	0	-
University	22 (100)	22 (95.7)	-4.3	-13 to 4	0 (0.0)	0 (0.0)	0.0	-	0 (0.0)	0 (0.0)	0	-
**Religion**												
Anglican	225 (91.5)	248 (98.8)	8.0	4 to 12	12 (4.9)	2 (0.8)	-83.7	-114 to -53	9 (3.7)	1 (0.4)	-89.2	-126 to -52
Roman Catholic	90 (96.8)	80 (97.6)	0.8	-4 to 6	1 (1.1)	2 (2.4)	118.2	-	2 (2.2)	0 (0.0)	-100.0	-
Pentecostal	97 (94.2)	98 (95.1)	1.0	-5 to 7	3 (2.9)	3 (2.9)	0.0	-27 to 27	3 (2.9)	2 (1.9)	-34.5	-62 to -7
Muslim	525 (91.6)	362 (95.3)	4.0	1 to 7	30 (5.2)	14 (3.7)	-28.9	-42 to -15	18 (3.1)	4 (1.1)	-64.5	-82 to -47
Other	17 (89.5)	10 (100)	11.7	-2 to 26	0 (0.0)	0 (0.0)	0.0	-	2 (10.5)	0 (0.0)	0	-
**Overall**	**954 (92.3)**	**798 (96.6)**	**4.6**	**3 to 7**	0 (0.0)	0 (0.0)	**-43.2**	**-53 to -33**	**33(3.3)**	0 (0.0)	**-75.8**	**-89 to-62**
