# Supplementary material for: The effect of community dialogues and sensitization on patient reporting of adverse events in rural Uganda: Uncontrolled before-after study
Source: PLoS One. 2019 May 9;14(5):e0203721. doi: 10.1371/journal.pone.0203721 (PMC6508596; doi:10.1371/journal.pone.0203721)
Supplement: S2 Table — Comparison of respondents’ having ever experienced ADEs by socio-demographic characteristics before and after the CDS program. A comparison of the potential to report adverse drug events in response to the question of whether participants had ever experienced one before and after. (PDF) [file pone.0203721.s005.pdf]

Comparison of respondents' having ever experienced ADEs by socio-demographic characteristics before and after CDS.

	Yes				No				Don't know			
	**Before (%)**	**After (%)**	**% age diff.**	**95% CI**	**Before (%)**	**After (%)**	**% age diff.**	**95% CI**	**Before (%)**	**After (%)**	**% age diff.**	**95% CI**
**Age group**												
15-24	51 (31.3)	43 (43.0)	37.4	18 to 57	108 (66.3)	57 (57)	-14.0	-29 to 1	4 (2.5)	0 (0.0)	-100.0	-
25-34	109 (35.9)	61 (42.1)	17.3	2 to 32	183 (60.2)	84 (57.9)	-3.8	-16 to 9	12 (3.9)	0 (0.0)	-100.0	-
35-44	80 (32.9)	61 (47.3)	43.8	28 to 60	159 (65.4)	67 (51.9)	-20.6	-34 to -7	4 (1.6)	1 (0.8)	-50.0	-76 to -24
45-54	47 (32.4)	43 (46.7)	44.1	24 to 64	96 (66.2)	47 (51.1)	-22.8	-40 to -6	2 (1.4)	2 (2.2)	57.1	31 to 83
55-64	36 (34.3)	13 (35.1)	2.3	-28 to 32	68 (64.8)	24 (64.9)	0.2	-22 to 22	1 (1.0)	0 (0.0)	-100.0	-
65+	26 (35.1)	16 (36.4)	3.7	-26 to 34	46 (62.2)	28 (63.6)	2.3	-20 to 25	2 (2.7)	0 (0.0)	-100.0	-
**Education**												
Primary	180 (33.8)	120 (42.4)	25.4	14 to 37	340 (63.9)	161 (56.9)	-10.9	-20 to -2	12 (2.3)	2 (0.7)	-69.6	-91 to -48
Secondary	109 (35.2)	73 (40.1)	13.9	0 to 28	194 (62.6)	109 (59.9)	-4.3	-16 to 7	7 (2.3)	0 (0.0)	-100.0	-
Tertiary	12 (52.2)	8 (40.0)	-23.4	-68 to 21	10 (43.5)	12 (60.0)	-86.2	-118 to -55	1 (4.3)	0 (0.0)	-100.0	-
University	10 (45.5)	13 (65.0)	42.9	16 to 70	12 (54.5)	7 (35.0)	-35.8	-73 to 1	0 (0.0)	0 (0.0)	0.0	-
**Religion**												
Anglican	89 (36.2)	66(38.6)	6.6	-9 to 22	152 (61.8)	104 (60.8)	-1.6	-14 to 11	5 (2.0)	1 (0.6)	-70.0	-98 to -42
Roman Catholic	23 (24.7)	20(41.7)	68.8	41 to 96	69 (74.2)	28 (58.3)	-21.4	-41 to -1	1 (1.1)	0 (0.0)	-100.0	-
Pentecostal	45 (43.7)	32(46.4)	6.2	-16 to 29	55 (53.4)	37 (53.6)	0.4	-20 to 21	3 (2.9)	0 (0.0)	-100.0	-
Muslim	189 (33)	115(45.6)	38.2	27 to 49	369 (64.4)	135 (53.6)	-16.8	-26 to -7	15 (2.6)	2 (0.8)	-69.2	-92 to -47
Other	3 (15.8)	4(57.1)	261.4	195 to 328	15 (78.9)	3 (42.9)	-45.6	-98 to 7	1 (5.3)	0 (0.0)	-100.0	-
**Overall**	**349 (20.5)**	**237(44)**	**114.6**	**137 to 217**	**660 (63.8)**	**307 (29.7)**	**-53.5**	**-84 to -3**	**25 (2.4)**	0 (0.0)	**-87.9**	**-50 to 21**
