# Supplementary material for: The effect of community dialogues and sensitization on patient reporting of adverse events in rural Uganda: Uncontrolled before-after study
Source: PLoS One. 2019 May 9;14(5):e0203721. doi: 10.1371/journal.pone.0203721 (PMC6508596; doi:10.1371/journal.pone.0203721)
Supplement: S3 Table — A comparison of the type of adverse events that the respondents would report before and after the CDS program. (PDF) [file pone.0203721.s006.pdf]

A comparison of the type of adverse events that the respondents would report before and after the CDS intervention

	Yes/Agree n (%)				No/Disagree n (%)				Don't know n (%)			
	**Before (%)**	**After (%)**	**% Diff**	**95% CI**	**Before (%)**	**After (%)**	**% Diff**	**95% CI**	**Before (%)**	**After (%)**	**% Diff**	**95% CI**
Uncertain or suspected ADEs	807 (78.0)	721 (87.3)	11.9	9 to 15	133 (12.9)	84 (10.2)	-20.9	-28 to -14	94 (9.1)	21 (2.5)	-72.5	-80 to -65
Certain negative ADEs	697 (67.4)	634 (76.7)	13.8	10 to 18	235 (22.7)	168 (20.3)	-10.6	-17 to -4	102 (9.9)	24 (2.9)	-70.7	-78 to -63
Serious reactions	797 (77.1)	756 (91.5)	18.7	16 to 21	143 (13.8)	57 (6.9)	-50.0	-57 to -43	94 (9.1)	13 (1.6)	-82.4	-90 to -75
Mild reactions	728 (70.4)	573 (69.4)	-1.4	-6 to 3	243 (23.5)	246 (29. 8)	26.8	20 to 33	63 (6.1)	7 (0.9)	-85.3	-93 to -78
Reactions to drugs which have been on market for a long period	718 (69.4)	649 (78.6)	13.3	10 to 17	223 (21.6)	146 (17.7)	-18.1	-24 to -12	93 (9.0)	31 (3.7)	-58.9	-65 to -52
Reactions to newly introduced drugs	770 (74.5)	706 (85.5)	14.8	11 to 18	147 (14.2)	73 (8.8)	-38.0	-46 to -31	117 (11.3)	47 (5.7)	-49.6	-57 to -42
Common or well-known reactions	639 (61.8)	483 (58.5)	-5.3	-10 to -1	285 (27.6)	315 (38.1)	38.0	32 to 44	110 (10.6)	28 (3.4)	-67.9	-75 to -61
Unexpected reactions	737 (71.3)	683 (82.7)	16	13 to 19	186 (18.0)	107 (12.9)	-28.3	-35 to -21	111 (10.7)	36 (4.4)	-58.9	-66 to -52
Possible interaction with other drugs	660 (63.8)	582 (70.5)	10.5	6 to 15	231 (22.3)	179 (21.7)	-2.7	-9 to 4	143 (13.8)	65 (7.9)	-42.8	-50 to -35
Reaction due to herbal medicine	573 (55.4)	520 (62.9)	13.5	9 to 18	370 (35.8)	279 (33. 8)	-5.6	-11 to 0.0	91 (8.8)	27 (3.3)	-62.5	-70 to -55
Reactions due to herbal & conventional medicine taken together	643 (62.2)	616 (74.6)	19.9	16 to 24	301 (29.1)	179 (21.7)	-25.4	-31 to -19	90 (8.7)	31 (3.7)	-57.5	-64 to -51

ADEs = adverse drug events
