# Supplementary material for: The effect of community dialogues and sensitization on patient reporting of adverse events in rural Uganda: Uncontrolled before-after study
Source: PLoS One. 2019 May 9;14(5):e0203721. doi: 10.1371/journal.pone.0203721 (PMC6508596; doi:10.1371/journal.pone.0203721)
Supplement: S1 Fig — Comparison of options for reaching community members with ADE messages as perceived by respondents and healthcare professionals. (PDF) [file pone.0203721.s007.pdf]

Fig 1: Comparison of options for reaching community members with ADE messages as perceived by respondents and healthcare professionals.

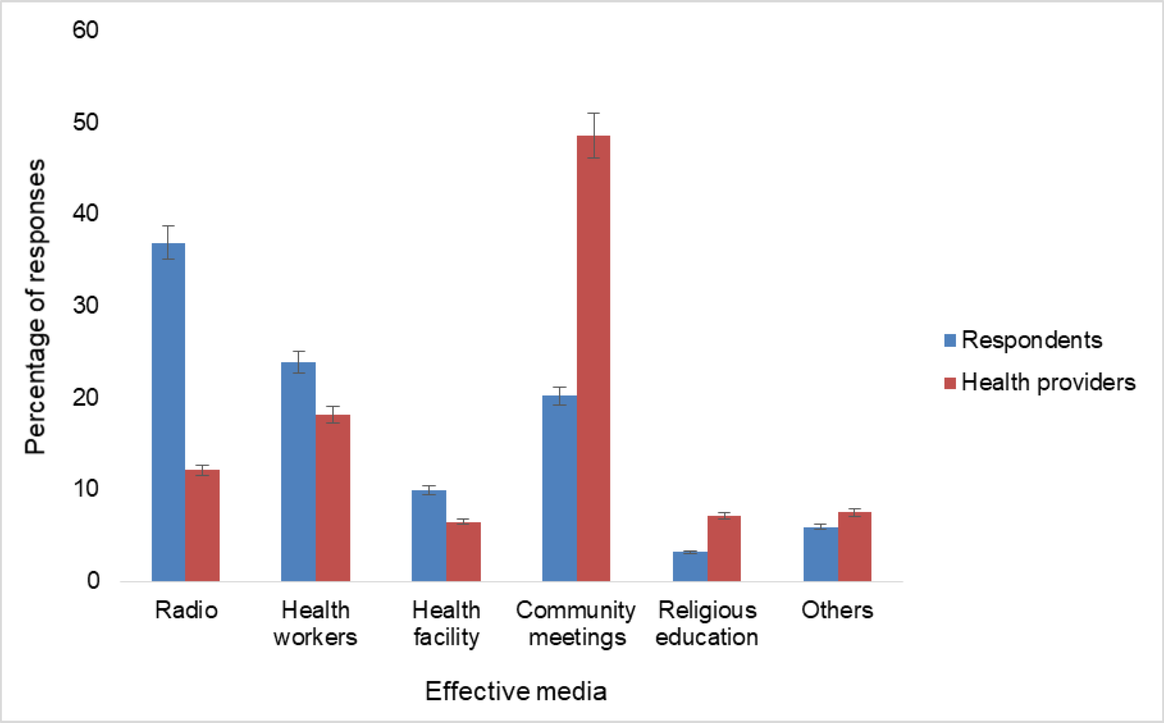

*Error bars represent 95% confidence intervals of the percentage responses of community members
